# Highly conserved ion binding sites are not all functionally relevant in mouse KCC4

**DOI:** 10.3389/fmolb.2025.1556250

**Published:** 2025-03-31

**Authors:** Lisa Becker, Jens Hausmann, Rieke Wellpott, Anna-Maria Hartmann

**Affiliations:** ^1^ Division of Neurogenetics, School of Medicine and Health Science, Carl von Ossietzky University of Oldenburg, Oldenburg, Germany; ^2^ Division of Anatomy, School of Medicine and Health Science, Carl von Ossietzky University of Oldenburg, Oldenburg, Germany; ^3^ Research Center Neurosensory Sciences, Carl von Ossietzky University of Oldenburg, Oldenburg, Germany

**Keywords:** KCC, large extracellular loop, site directed mutagenesis, protein conformation, ion binding sites

## Abstract

**Introduction:**

The potassium chloride cotransporter 4 (KCC4) is expressed in various tissues and plays an important role in distal renal acidification and hearing development. Although KCCs transport K^+^ and Cl^−^ in a 1:1 stoichiometry, two Cl^−^ coordination sites were indicated via cryo-electron microscopy (CryoEM).

**Methods:**

In a comprehensive analysis, we analyzed here the consequences of point mutation of residues coordinating potassium, and chloride in the first (Cl_1_) and second (Cl_2_) coordinating site in KCC4 using Tl^+^ based flux measurements.

**Results:**

Surprisingly, not all highly conserved coordination sites in KCC4 are essential. Three out of five residues (N^131^, Y^216^, and T^432^) are functionally relevant for potassium coordination. For chloride coordination in Cl_1_, all three residues (G^134^, V^135^, and I^136^) are important, whereas three out of four residues (G^433^, M^435^, and Y^589^) are relevant for chloride binding in Cl_2_. As all ion coordination sites are important in KCC2, this indicates that there is a certain flexibility in the stringency of ion coordination in KCC4. One possible reason for the different relevance of ion coordination sites could be the large extracellular loop (LEL). The LEL is structured differently within KCCs and is directly linked to the transmembrane domain (TM) 6, where most of the coordination sites reside. Substitution of ion coordination sites in the KCC2_2-4-2_ chimera, in which the LEL from mouse KCC4 is exchanged with the LEL of rat KCC2, have the same effect as substitutions in rat KCC2. An exception is the substitution of the potassium coordination site I^111^ in TM1, which shows enhanced activity in the KCC2_2-4-2_ chimera compared to the impaired activity in rat KCC2 and not affected activity in mouse KCC4.

**Conclusion:**

Thus, the different relevance of the ion coordination sites between KCC2 and KCC4 cannot be attributed solely to the different structured LEL; other structural elements must also be involved here.

## Introduction

The potassium chloride cotransporters (KCCs) are secondary active membrane proteins, that are important in cell volume regulation, transepithelial transport, and regulation of the intracellular chloride concentration ([Cl^−^]) (Boettger et al., 2002; Gamba, 2005). They belong to the so-called cation chloride cotransporter (CCC) family, that harbors the electroneutral sodium independent K^+^ and Cl^−^ outward cotransporters (KCCs), the sodium dependent Na^+^, K^+^, 2 Cl^-^ inward cotransporters (NKCCs), and the Na^+^, Cl^−^ inward cotransporter NCC (Gamba, 2005; Hartmann et al., 2014). This protein family also includes the polyamine transporter CCC9 and the transport-inactive cation-interacting protein CIP1 (Gamba, 2005; Hartmann et al., 2014; Wenz et al., 2009; Daigle et al., 2009). KCCs are subdivided into four paralogous KCC1 to KCC4 (Hartmann et al., 2014). Among these, KCC4 encoded by the *SLC12A7* gene (Gamba, 2005; Marcoux et al., 2017; Mount et al., 1999), is expressed in various tissues, including the heart, kidney, lung, liver, gastric glands, nervous system, and inner ear (Marcoux et al., 2017; Mount et al., 1999; Karadsheh et al., 2004; Fujii et al., 2009; Garneau et al., 2019; Velazquez and Silva, 2003). Mice lacking *SLC12A7* suffer from progressive hearing loss and renal tubular acidosis (Boettger et al., 2002). These conditions are caused by disrupted K^+^ recycling by the supporting Deiter’s cell in the cochlea and impaired Cl^−^ recycling by α-intercalated cells in the kidney’s distal nephron, respectively (Boettger et al., 2002; Marcoux et al., 2017; Garneau et al., 2019). To date, no human pathogenic variants in KCC4 have been found, that have an effect on the transport activity of KCC4 (Marcoux et al., 2017). In the phylogenetically closely related KCC2, human pathogenic variants were not known for a long time. Only in recent years human pathogenic variants in KCC2 were investigated, that are associated with epilepsy, autism-spectrum disorders and schizophrenia (Kahle et al., 2014; Merner et al., 2015; Stödberg et al., 2015; Fukuda and Watanabe, 2019).

**FIGURE 1:**
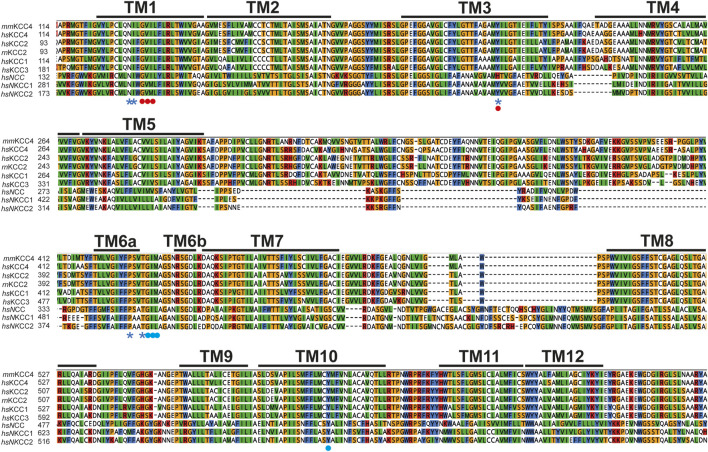
Evolutionary conversation of K^+^ and Cl^−^ binding site. Multialignment of TM1 to TM10 of mouse KCC4 (NP_035520.1), rat KCC2b (NP_599190.1), human KCC1 (NP_005063.1), KCC2 (NP_065759.1), KCC3 (NP_598408.1), KCC4 (NP_006589.2), NKCC1 (NP_001037.1), NKCC2 (NP_001171761.1), and NCC (NP_000330.2) was generated using Clustal Omega in Geneious. The TMs, K^+^, and Cl^−^ binding sites were annotated according to the CryoEM based 3-dimensional structure of *mm*KCC4 (PDB: 6UKN) (Reid et al., 2020). K^+^ binding sites located in TM1 (N and I), TM3 (Y), and TM6 (P and T) are marked with a blue asterisk. Cl^−^ binding sites in Cl_1_ located in TM1 (G, V, and I) are marked with red dots. Cl^−^ binding sites in Cl_2_ located in TM6 (G, I, and M) and TM10 (Y) are marked with blue dots. Abbreviations used are as follows: *mm*, *Mus musculus, rn, Rattus Norvegicus* and *hs*, *Homo sapiens*.

**FIGURE 2:**
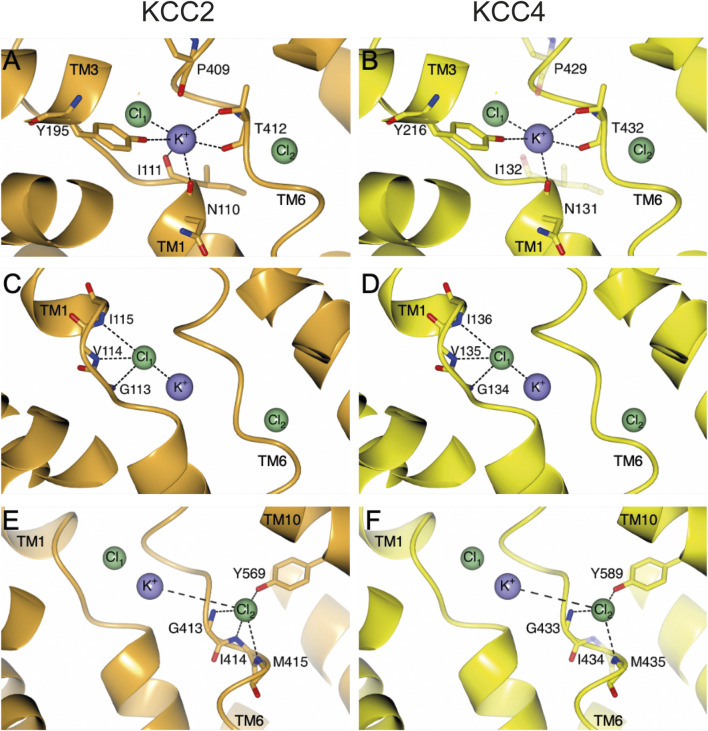
Structural overview of ion coordination in KCC2 (orange) and KCC4 (yellow). The structural overview is based on the PDB entry 7D99 (Xie et al., 2020). Ion coordination sites Cl_1_ **(A, B)**, K^+^ **(C, D)** and Cl_2_ **(E, F)** with their respective residues that are involved in ion coordination are depicted with side and/or main chain. Amino acids (*mm*KCC4^I132S^, *mm*KCC4^P429H^ and *mm*KCC4^I434E^), which do not significantly influence the transport activity of *mm*KCC4, when substituted, are illustrated as transparent residues in their native form. Intramolecular interactions are indicated as dashed lines. Cl^−^ ions are shown as green spheres and K^+^ ion is shown as purple sphere. Structural depictions have been generated with CCP4mg (McNicholas et al., 2011).

On the structural level, KCCs consist of 12 transmembrane domains (TMs) and a large glycosylated extracellular loop (LEL) is located between TM5 and TM6 (Gamba, 2005; Mount et al., 1999; Payne et al., 1996; Hartmann and Nothwang, 2015; Weng et al., 2013). The N- and C-terminus are located intracellularly (Gamba, 2005; Mount et al., 1999; Payne et al., 1996; Hartmann and Nothwang, 2015). The aforementioned TMs are important for ion translocation and the termini have a regulatory role (Gamba, 2005; Marcoux et al., 2017; Hartmann and Nothwang, 2015; Chi et al., 2021; Chew et al., 2021; Zhang et al., 2021; Reid et al., 2020; Rinehart et al., 2009; Weber et al., 2014; Hartmann and Nothwang, 2022; Bergeron et al., 2006; Mercado et al., 2006). The N-terminus has an auto-inhibitory function in KCCs locking the transporter in the inward facing state to prevent ion translocation (Chi et al., 2021; Zhang et al., 2021; Xie et al., 2020). In recent years, three-dimensional structures of KCCs, NKCC1 and NCC from different species were determined by single-particle cryogenic electron microscopy (CryoEM), in which the ion coordination sites were determined (Chi et al., 2021; Chew et al., 2021; Zhang et al., 2021; Reid et al., 2020; Xie et al., 2020; Chew et al., 2019; Liu et al., 2019). The potassium binding site is formed by N^131^ and I^132^ of TM1, Y^216^ in TM3 and P^429^ as well as T^432^ in TM6 (Figures 1, 2) (annotated according to *mus musculus* KCC4 (*mm*KCC4)) (Chi et al., 2021; Zhang et al., 2021; Reid et al., 2020; Chew et al., 2019; Liu et al., 2019). To test the relevance of the ion binding sites on the function of the transporters, they were mutated in various CCCs and then functionally analyzed. Substitution of these residues in KCC1 (tyrosine in TM3), KCC2 (asparagine and isoleucine in TM1, tyrosine in TM3, and proline and threonine in TM6), KCC3 (tyrosine in TM3 and threonine in TM6), KCC4 (asparagine in TM1 and tyrosine in TM3), and NKCC1 (tyrosine in TM3 and proline and threonine in TM6) diminished the transport activities (Zhang et al., 2021; Reid et al., 2020; Chew et al., 2019; Liu et al., 2019; Hartmann et al., 2021). Two chloride coordination sites were delineated in NKCCs in cryo-EM structures in the presence of a 1 Na^+^: 1K^+^: 2 Cl^-^ stoichiometric ratio (Chew et al., 2019; Neumann et al., 2021). Unexpectedly, these two chloride coordination sites are also present in KCCs, although KCCs transport K^+^ and Cl^−^ ions in a 1:1 stoichiometry (Chi et al., 2021; Zhang et al., 2021; Reid et al., 2020; Xie et al., 2020; Liu et al., 2019; Becker et al., 2023). The first chloride coordination site (Cl^−^_1_), consists of main-chain interactions of G^134^, V^135^ and I^136^ in TM3 (Figures 1, 2) (Chi et al., 2021; Chew et al., 2021; Zhang et al., 2021; Reid et al., 2020; Liu et al., 2019; Neumann et al., 2021; Becker et al., 2023; Nan et al., 2022). The second chloride coordination site (Cl_2_) is also formed by main chain interactions of G^433^, I^434^, M^435^ in TM6, and side chain of Y^589^ in TM10 (Figures 1, 2) (Chi et al., 2021; Chew et al., 2021; Zhang et al., 2021; Reid et al., 2020; Liu et al., 2019; Neumann et al., 2021; Nan et al., 2022). Substitutions of all chloride coordination sites in KCC2 (Cl_1_ and Cl_2_), and tyrosine in TM10 (Cl_2_) in KCC1, KCC3, KCC4, NKCC1, and NCC, resulted in diminished or abolished activities, indicating that Cl_1_ and Cl_2_ are both important for transporter function (Zhang et al., 2021; Reid et al., 2020; Chew et al., 2019; Liu et al., 2019; Neumann et al., 2021; Becker et al., 2023; Nan et al., 2022).

Although the ion binding sites in CCCs are highly conserved, the potassium and chloride affinities differ (Gamba, 2005; Delpire and Guo, 2020). The question therefore arises which other structural elements could have an influence on the ion affinities. One possibility is the LEL. The LEL is directly linked to TM6, in which most of the ion coordination sites reside (Zhang et al., 2021; Reid et al., 2020; Hartmann et al., 2021; Becker et al., 2023) (Figure 1). Three-dimensional reconstructions of the LEL of KCCs shows that the structural elements within the LEL vary among KCCs (Chi et al., 2021; Reid et al., 2020; Liu et al., 2019). In KCC1, the LEL consists of two pairs of antiparallel ß-strands and four short helices (Liu et al., 2019). In KCC2, two antiparallel ß-strands and three short helices are present (Chi et al., 2021; Hartmann and Nothwang, 2022), whereas in KCC4, three short-stranded antiparallel ß-strands and three short helices exist (Reid et al., 2020). What they have in common are four highly conserved cysteines that form disulfide bridges between C^308^-C^323^ and C^343^-C^352^ (annotation according to *mm*KCC4) and thus stabilize the LEL (Chi et al., 2021; Reid et al., 2020; Xie et al., 2020; Hartmann et al., 2010; Chi et al., 2020). Substitutions of all four cysteines inactivate KCC2, indicating that the cysteines are important for transporter function (Hartmann et al., 2010). However, substitution of analogues cysteines in KCC4 have no effect on its activity (Hartmann et al., 2010). Furthermore, swapping the LEL of mouse KCC4 on rat KCC2b (KCC2_2-4-2_ chimera) has no effect on the KCC2 activity, whereas swapping the LEL of rat KCC2b on mouse KCC4 (KCC4_4-2-4_ chimera) keeps KCC4 transport inactive (Hartmann et al., 2010). These analyses show that the LEL in KCCs is differentially organized and differentially affects the functionality of the transporters (Hartmann et al., 2010). Thus, it is possible that the different structural organization of the LEL could have a direct effect on ion coordination.

Here, we comprehensively analyze the relevance of all ion coordination sites in *mouse* KCC4. Our analyses revealed, that three (N^131^, Y^216^, and T^432^) out of five residues are necessary for K^+^ coordination (Figure 2B). For chloride coordination, all three residues in Cl_1_ (G^134^, V^135^, and I^136^) and three (G^433^, M^435^, and Y^589^) out of four residues in Cl_2_ are functionally relevant (Figures 2D, F). These results are surprising, as the closest related transporter KCC2 requires all ion coordination sites (Figures 2A, C, E) (Hartmann et al., 2021; Becker et al., 2023). By using the KCC2_2-4-2_ chimera we show that the different relevance of most ion coordination sites, except for I^111^ (annotation according to rat KCC2b), cannot be explained by the different structural organization of the LEL. Therefore, other structural elements are likely to have a further influence.

## Material and methods

### Graphical representation

We used the protein data bank (PDB) entry 7D99 (Xie et al., 2020) for analysis and structural representation. Figures have been generated with CCP4mg (McNicholas et al., 2011). Effects of amino acid substitutions have been displayed in Coot (Emsley and Cowtan, 2004) and secondary-structure matching (Krissinel and Henrick, 2004) was used with the PDB entry 6ukn (Reid et al., 2020) and 6m23 (Chi et al., 2021) for KCC4 and KCC2, respectively. We additionally used AlphaFold3 (Abramson et al., 2024) to validate the functional consequences of the mutant variant N^131S^ and G^443A^ by structural prediction. Here, the sequence of the uniprot entry Q9Y666-1 for human KCC4 has been used after a virtual mutagenesis of residue position 131 from asparagine to serine, for the residue position 433 from glycine to alanine, respectively.

### Construction of the expression clones

For site-directed mutagenesis, *rattus norvegicus* KCC2b (*rn*KCC2b, NM_134363.1), *mus musculus* KCC4 (*mm*KCC4, NM_011390) and the chimera KCC2_2-4-2_ were used as starting constructs. The generation of these constructs is described in Hartmann et al. (2010). The chimera KCC2_2-4-2_ consists of the *rn*KCC2b backbone and the extracellular loop of *mm*KCC4. Site-directed mutagenesis was performed according to the QuikChange mutagenesis system (Stratagene Heidelberg, Germany). Supplementary Tables 1, 2 contain forward oligonucleotides of all generated mutations. The plasmids were prepared using the Genopure plasmid Maxi kit (Merck). All generated clones were verified by sequencing (Eurofins Genomics, Berlin, Germany).

### Cell culturing

Human embryonic kidney cells (HEK293) were transiently transfected using Turbofect (Fermentas, Schwerte, Germany) prior to the immunocytochemistry and measurements of the transport activity of the constructs. To do so, cells were seeded in a 6-well plate and incubated for 24 h. Four hours prior to transfection the DMEM medium was replaced. For transfection 150 µL Opti-MEM (Invitrogen, Karlsruhe, Germany), 6 µL Turbofect, and 3–4.5 μg/μL DNA were mixed and incubated for 20 min at room temperature. Afterwards, the mixture was applied to the cells followed by shaking at 300 rpm for 10 min. After incubation for 24 h, the HEK293 cells were plated at a concentration of 1 × 10^5^ cells/well in a 0.1 mg/mL poly-L-lysine-coated black-well 96 well culture dish (Greiner Bio-One, Frickenhausen, Germany) for K^+^-Cl^-^ cotransporter activity measurements. Three technical replicates have been seeded for each transfected construct. The constructs were transfected separately to generate independent biological replicates. To determine the transfection rate by immunocytochemical analysis, the remaining cells were plated on 0.1 mg/mL poly-L-lysine-coated glass coverslips. After ∼18 h, coverslips were proceeded for immunocytochemical analysis to determine transfection rates, which were routinely between 20% and 30%.

### Immunocytochemistry

All steps were performed at room temperature. For fixation, the HEK293 cells, which were grown on poly-L-lysine-coated coverslips, were treated with 4% paraformaldehyde in 0.2 M phosphate buffer (pH 7.4) and incubated for 10 min. Afterwards, the cells were washed three times with PBS. Blocking solution (2% bovine serum albumin and 10% goat serum in PBS) was applied for 30 min. Then, the primary antibody solution (anti-KCC2 N1-12, 1:1000, Neuromab, California, USA; anti-KCC4; 1:250, Invitrogen, Karlsruhe, Germany) diluted in carrier solution (0.3% Triton X-100, 1% bovine serum albumin, and 1% goat serum in PBS) was applied and incubated for 1 h. Followed by triple washing with PBS again. Afterwards, the secondary antibody solution was applied (Alexa Flour 488 goat anti-mouse, 1:1000, Thermo Fisher Scientific, Bremen, Germany; Alexa Flour 488 goat anti-rabbit, 1:500, Thermo Fisher Scientific, Bremen, Germany) and incubated for an additional hour in the dark. Again, cells were washed three times with PBS, followed by complete drying. The dried coverslips were mounted onto glass slides with Mowiol (Roth, Karlsruhe, Germany) and 4,6-diamidine-2- phenylindole (1:1000 dilution; Roth). Images were taken using an Olympus fluorescence microscope (Olympus BX63).

### Determination of the K^+^-Cl^-^ cotransport activity

Transport activities of KCC2 and KCC4 were determined by Cl^−^ dependent uptake of Tl^+^ in HEK293 cells as described previously (Becker et al., 2023; Hartmann et al., 2010; Hartmann et al., 2009). For initiation of the flux measurement, the DMEM medium was replaced by 80 µL of hypotonic preincubation buffer (100 mM N-methyl- D-glucamine-chloride for KCC2 or 65 mM for KCC4, 5 mM Hepes, 5 mM KCl, 2 mM CaCl_2_, 0.8 mM MgSO_4_, 5 mM glucose, pH 7.4) with 2 µL Flouzin-2 AM dye (Invitrogen) and 0.2% (w/v) Pluronic F-127 (Invitrogen) and incubated for 48 min at room temperature in the dark. The cells were then washed three times with preincubation buffer and incubated for 15 min in the dark with preincubation buffer plus 0.1 mM ouabain to block the activity of the Na^+^/K^+^ ATPase. Afterwards, the 96-well plate was placed into the fluorometer (Fluoreskan FL, Thermo Fisher Scientific). Each well was injected with 40 µL of 5x Thallium stimulation buffer (12 mM Tl_2_SO_4_, 100 mM/65 mM N-methyl-D-glucamine respectively, 5 mM Hepes, 2 mM KCl, 2 mM CaCl_2_, 0.8 mM MgSO_4_, 5 mM glucose, pH 7.4). The fluorescence was measured in a kinetic-dependent manner (excitation 485 nm, emission 538 nm, one frame in 6 s in a 200 s period) across the entire cell population in a single well. The transport activity was calculated by using linear regression of the initial values of the slope of Tl^+^-stimulated fluorescence increase. By setting the slope of the KCC2^WT^, KCC4^WT^ or KCC2_2-4-2_ chimeric constructs as 100%, the activity of the mutants is transformed into a percentage of the activity and normalized. Normalization is needed to subtract naturally occurring fluctuations from the fluorescence increase.

### Statistical analysis

For statistical analysis, a two-sample Student’s t-test was used to test the normalized transport activities of the respective mutants against control samples (*mm*KCC4^WT^, *rn*KCC2b^WT^, KCC2b_2-4-2_ and mock). If the standard deviation differs by more than a factor of 2, the Welch’s t-test was used (Welch, 1938). The number of degrees of freedom was deflated according to the size of independent preparations to avoid pseudo-replication. P-values were corrected, and the false discovery rate was controlled by using the Benjamini–Hochberg method (Benjamini and Hochberg, 1995). By choosing p values of <0.01 the change of type I error was reduced.

## Results

### Potassium binding sites in KCC4

**FIGURE 3:**
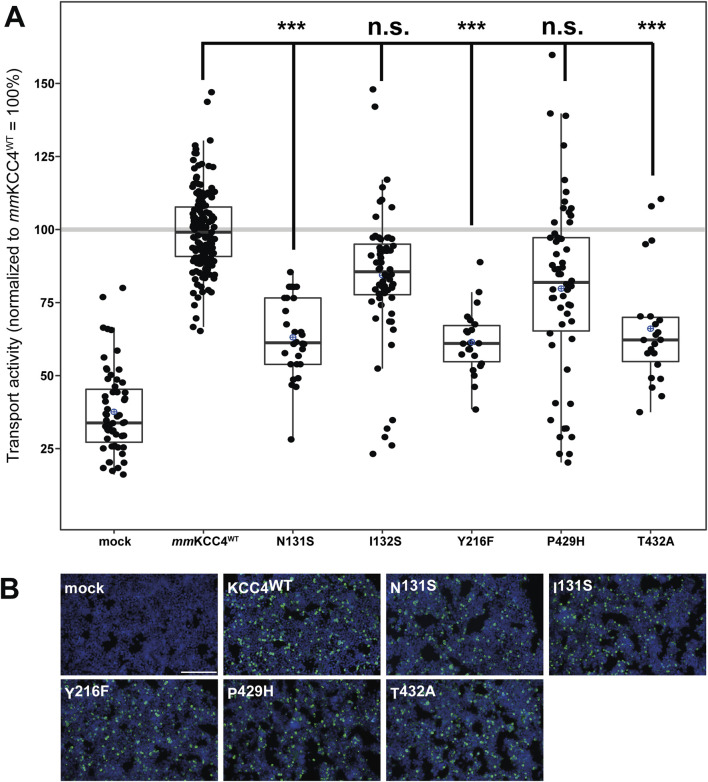
Substitutions of potassium binding sites differentially impair KCC4 function. HEK293 cells were transiently transfected with *mm*KCC4^WT^ or *mm*KCC4 variants with mutations in the potassium binding site. Cells were then seeded in parallel for Tl^+^ flux measurements and immunocytochemistry. **(A)** Tl^+^ flux measurements were performed to determine the transport activity. The predicted K^+^ residue mutants *mm*KCC4^N131S^ (63% ± 13%, p = 1.43 × 10^−18^), *mm*KCC4^Y216F^ (62% ± 11%, p = 1.33 × 10^−17^) and *mm*KCC4^T432A^ (66% ± 20%, p = 3.14 × 10^−14^) showed diminished KCC4 transport activity compared to KCC4^WT^ (100% ± 14%), whereas *mm*KCC4^I132S^ (90% ± 31%, p = 0.015) and KCC4^P429H^ (87% ± 41%, p = 0.018) do not impair KCC4 transport activity. The graph represents the data of at least six independent measurements including three technical replicates, normalized to *mm*KCC4^WT^ (100%). The figure shows the statistical comparison between *mm*KCC4^WT^ and the mutants (p ≥ 0.01: n.s., p < 0.001:***). **(B)** Immunocytochemistry was used to monitor the transfection rate of the *mm*KCC4 variants (green) and cell staining by DAPI (blue). Representative immunocytochemical images were used for the biological replicates. The scale bar represents 200 µm.

CryoEM structures of cation chloride cotransporters (CCCs) revealed a coordination of the potassium ion via five highly conserved amino acid residues located in TM 1 (asparagine and isoleucine), TM3 (tyrosine), and TM6 (proline and threonine) (Chi et al., 2021; Zhang et al., 2021; Reid et al., 2020; Chew et al., 2019; Liu et al., 2019; Neumann et al., 2021; Yang et al., 2020) in agreement with functional analyses in KCC1 to KCC4 and NKCC1 (Zhang et al., 2021; Reid et al., 2020; Chew et al., 2019; Liu et al., 2019; Hartmann et al., 2021). However, a detailed functional analysis of the potassium binding site in KCC4 is missing (Reid et al., 2020). To confirm the functional relevance of residues coordinating potassium, we mutated the corresponding sites into residues of a similar size but different chemical properties. This results in the followingmouse KCC4 mutations: *mm*KCC4^N131S^, *mm*KCC4^I132S^, *mm*KCC4^Y216F^, *mm*KCC4^P429H^ and *mm*KCC4^T432A^. All five mutants showed transfection rates in HEK293 cells equal to that of *mm*KCC4^WT^ (Figure 3B). Tl^+^ based flux measurements revealed significantly reduced transport activities for three out of five mutants by an average of 63% ± 14% for *mm*KCC4^N131S^ (p = 1.43 × 10^−18^), 62% ± 11 for *mm*KCC4^Y216F^ (p = 1.33 × 10^−17^), and 66% ± 20 for *mm*KCC4^T432A^ (p = 3.14 × 10^−14^), compared with *mm*KCC4^wt^ (100% ± 14%). Surprisingly, mutation of *mm*KCC4^I132S^ (90% ± 31%, p = 0.015) and *mm*KCC4^P429H^ (87% ± 41%, p = 0.018) do not significantly impair KCC4 transport activity (Figure 3; Table 1). Thus, the only binding site residues found to be functionally relevant for potassium coordination in KCC4 are N^131^ in TM1, Y^216^ in TM3, and T^432^ in TM6.

**TABLE 1:** Transport activity of K^+^ mutations in *mm*KCC4.

Construct	Mean ± SD	Significance in comparison to *mm*KCC4^WT^	Significance in comparison to mock
Mock	38% ± 15%	***	-
*mm*KCC4^WT^	100% ± 14%	-	***
N131S	63% ± 13%	***	***
I132S	90% ± 31%	n.s.	***
Y216F	62% ± 11%	***	***
P429H	87% ± 41%	n.s.	***
T432A	66% ± 20%	***	***

Abbreviations used are as follows: for: *mm, mus musculus*, p ≥ 0,01: n.s., p < 0.001: ***.

### Cl^−^ binding sites in KCC4

**FIGURE 4:**
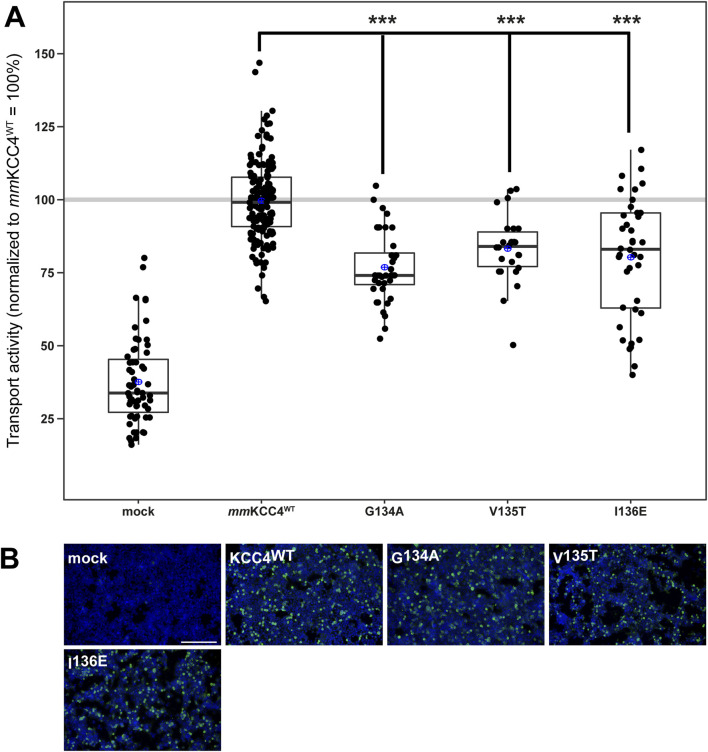
Substitutions of the chloride binding sites in Cl_1_ impair KCC4 transport activity. HEK293 cells were transiently transfected with *mm*KCC4^WT^ or *mm*KCC4 variants with mutations in the chloride binding site 1. Cells were then seeded in parallel for Tl^+^ flux measurements and immunocytochemistry. **(A)** Tl^+^ flux measurements were performed to determine the transport activity. The Tl^+^ flux measurements showed that *mm*KCC4^G134A^ (77% ± 12%, p = 1.04 × 10^−13^), *mm*KCC4^V135T^ (83% ± 12%, p = 4.38 × 10^−7^) and *mm*KCC4^I136E^ (80% ± 20%, p = 3.54 × 10^−5^) resulted in diminished KCC4 transport activity compared to *mm*KCC4^wt^ (100% ± 14%). The graph represents the data of at least five independent measurements, including three technical replicates, normalized to *mm*KCC4^WT^. The figure shows the statistical comparison between *mm*KCC4^WT^ and the mutants (p < 0.001:***) **(B)** Immunocytochemistry was used to monitor the transfection rate of the *mm*KCC2^WT^ variants (green) and cell staining by DAPI (blue). Representative immunocytochemical images were used for the biological replicates. The scale bar represents 200 μm.

CryoEM structures of CCCs also revealed that two chloride binding sites are present in addition to the potassium binding site and that these are also highly conserved among CCCs (Chi et al., 2021; Zhang et al., 2021; Reid et al., 2020; Xie et al., 2020; Liu et al., 2019; Neumann et al., 2021; Becker et al., 2023; Yang et al., 2020). The chloride ion in the first binding site (Cl_1_) is coordinated by glycine, valine, and isoleucine in TM1 (Zhang et al., 2021; Reid et al., 2020; Becker et al., 2023). All three Cl_1_ binding sites were substituted as follows: *mm*KCC4^G134A^, *mm*KCC4^V135T^, *mm*KCC4^I136E^. These mutants showed transfection rates in HEK293 cells equal to that of *mm*KCC4 (Figure 4B). The Cl_1_ binding site mutants *mm*KCC4^G134A^ (77% ± 12%, p = 1.04 × 10^−13^), *mm*KCC4^V135T^ (83% ± 12%, p = 4.38 × 10^−7^), and *mm*KCC4^I136E^ (80% ± 12%, p = 3.54 × 10^−5^) resulted in diminished KCC4 transport activity compared to *mm*KCC4^wt^ (100% ± 14%) (Figure 4A; Table 2). These results confirm the importance of all three chloride binding sites in Cl_1_ in KCC4.

**TABLE 2:** Transport activity of Cl_1_ mutations in *mm*KCC4.

Construct	Mean ± SD	Significance in comparison to *mm*KCC4^WT^	Significance in comparison to mock
Mock	38% ± 15%	***	-
*mm*KCC4^WT^	100% ± 14%	-	***
G134A	77% ± 12%	***	***
V135T	83% ± 12%	***	***
I136E	80% ± 20%	***	***

Abbreviations used are as follows: *mm, mus musculus,* p ≥ 0.01: n.s., p < 0.001: ***.

**FIGURE 5:**
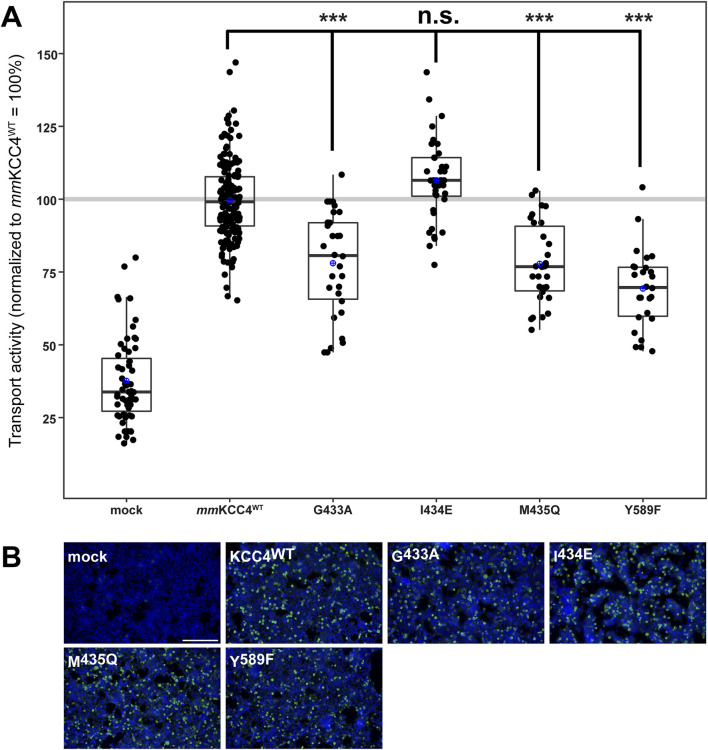
Substitutions of chloride binding sites in Cl_2_ differentially impair KCC4 function. HEK293 cells were transiently transfected with *mm*KCC4^WT^ or *mm*KCC4 variants with mutations in the chloride binding site 2. Cells were then seeded in parallel for Tl^+^ flux measurements and immunocytochemistry. **(A)** Tl^+^ flux measurements were performed to determine the transport activity. The Cl_2_ residue mutants *mm*KCC4^G433A^ (78% ± 18%, p = 3.7 × 10^−10^), *mm*KCC4^M435Q^ (78% ± 14%, p = 8.4 × 10^−11^) and *mm*KCC4^Y589F^ (69% ± 14%, p = 1.16 × 10^−14^) resulted in reduced KCC4 activity compared to *mm*KCC4^WT^ (100% ± 14%). The mutation *mm*KCC4^I434E^ (106% ± 14%, p = 0.012) did not alter KCC4 transport activity. The graph represents the data of at least five independent measurements, including three technical replicates, normalized to *mm*KCC4^WT^. The figure shows the statistical comparison between *mm*KCC4^WT^ and the mutants (p ≥ 0,01: n.s., p < 0.001:***) **(B)** Immunocytochemistry was used to monitor the transfection rate of the KCC4 variants (green) and cell staining by DAPI (blue). Representative immunocytochemical images were used for the biological replicates. The scale bar represents 200 µm.

To analyse the importance of the second chloride binding site (Cl_2_), which comprises the highly conserved residues in TM6 (glycine, isoleucine, and methionine) and TM10 (tyrosine) (Chi et al., 2021; Zhang et al., 2021; Reid et al., 2020; Xie et al., 2020; Liu et al., 2019; Neumann et al., 2021; Becker et al., 2023; Yang et al., 2020), we generated the following *mm*KCC4 mutants: *mm*KCC4^G433A^, *mm*KCC4^I434E^, *mm*KCC4^M435Q^, and *mm*KCC4^Y589F^. All four mutants showed transfection rates in HEK293 cells equal to that of *mm*KCC4^WT^ (Figure 5B). Three out of four mutants resulted in significantly diminished KCC4 transport activities by an average of 78% ± 18% for *mm*KCC4^G433A^ (p = 3.7 × 10^−10^), 78% ± 14% for *mm*KCC4^M435Q^ (p = 8.4 × 10^−11^), and 69% ± 14% for *mm*KCC4^Y589F^ (p = 1.16 × 10^−14^) compared to *mm*KCC4^wt^ (100% ± 14%) (Figure 5A; Table 3). Substitution of I^434^ with glutamine (106% ± 14%, p = 0.012) did not impair KCC4 transport activity (Figure 5A; Table 3). Therefore, the only binding site residues found to be functionally relevant for chloride coordination in Cl_2_ are G^433^ and M^435^ in TM6, and Y^589^ in TM10. These analyses also show that both chloride binding sites are required for KCC4-mediated transport.

**TABLE 3:** Transport activity of Cl_2_ mutations in *mm*KCC4.

Construct	Mean ± SD	Significance in comparison to *mm*KCC4^WT^	Significance in comparison to mock
Mock	38% ± 15%	***	-
*mm*KCC4^WT^	100% ± 14%	-	***
G433A	78% ± 18%	***	***
I434E	106% ± 14%	n.s.	***
M435Q	78% ± 14%	***	***
Y589F	69% ± 14%	***	***

Abbreviations used are as follows: *mm, mus musculus,* p ≥ 0.01: n.s., p < 0.001: ***.

### Ion binding sites in the KCC2_2-4-2_ chimera

**FIGURE 6:**
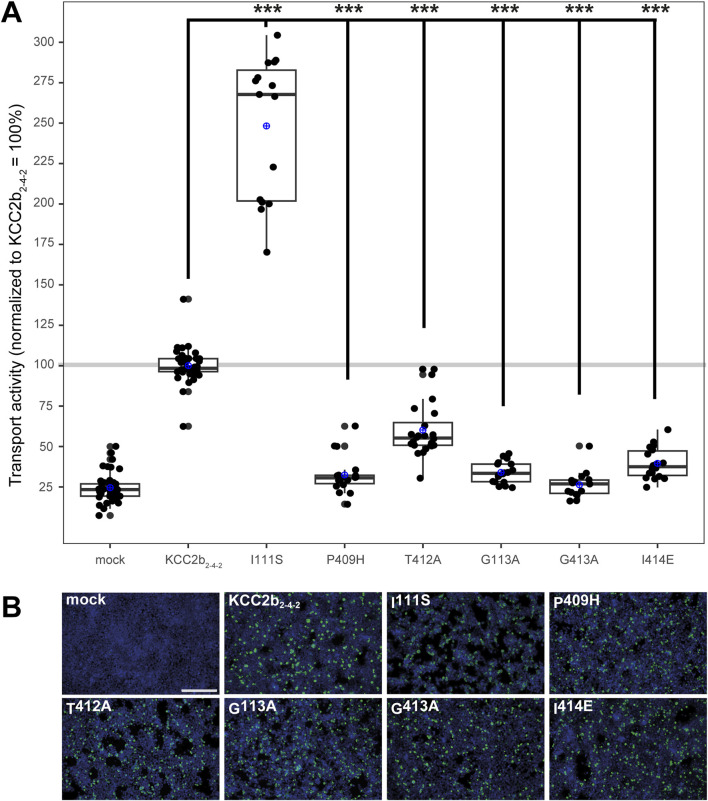
Substitutions of selected potassium and chloride binding sites affect KCC2_2-4-2_ chimera function. HEK293 cells were transiently transfected with KCC2_2-4-2_ variants with mutations in the potassium and chloride binding sites. Cells were then seeded in parallel for Tl^+^ flux measurements and immunocytochemistry. **(A)** Tl^+^ flux measurements were performed to determine the transport activity. The K^+^ binding site mutants KCC2_2-4-2_^P409H^ (33% ± 12%, p = 0.0086) abolished the transport activity compared to mock transfected cells (25% ± 9%), whereas KCC2_2-4-2_^T412A^ (60% ± 17%, p = 1.38 × 10^−6^) resulted in a diminished transport activity compared to KCC2_2-4-2_ (100% ± 10%). Contrary, KCC2_2-4-2_^I111S^ (248% ± 44%, p = 0.0002) resulted in an increased transport activity compared to KCC2_2-4-2_. The Cl_1_ site mutant KCC2_2-4-2_^G113A^ (34% ± 7%, p = 2.79 × 10^−14^) and the Cl_2_ site mutant KCC2_2-4-2_^I414E^ (40% ± 10%, p = 6.05 × 10^−14^) resulted in diminished transport activity compared to KCC2_2-4-2_ (100% ± 10%), whereas the Cl_2_ site mutant KCC2_2-4-2_^G413A^ (26% ± 8%, p = 0.46) resulted in abolished transport activities compared to mock transfected cells (25% ± 9%). The graph represents the data of at least five independent measurements, including three technical replicates, normalized to KCC2_2-4-2_. The figure shows the statistical comparison between KCC2_2-4-2_ and the mutants (p < 0.001: ***) **(B)** Immunocytochemistry was used to monitor the transfection rate of the KCC2 variants (green) and cell staining by DAPI (blue). Representative immunocytochemical images were used for the biological replicates. The scale bar represents 200 µm.

Although more ion coordination sites were detected in *mm*KCC4 by CryoEM (Reid et al., 2020), not all of them are functionally relevant. This differs from *rn*KCC2b, in which all detected coordination sites are essential (Hartmann et al., 2021; Becker et al., 2023). Due to the different functional relevance of the ion binding sites between KCC2 and KCC4 (Reid et al., 2020; Xie et al., 2020; Hartmann et al., 2010; Chi et al., 2020), we further investigated which structural elements could explain these differences. One possibility is the different structure of the LEL, which has different effects on the functionality of the transporters (Chi et al., 2021; Reid et al., 2020; Xie et al., 2020; Liu et al., 2019; Hartmann et al., 2010) and is directly connected to TM6, in which many ion binding sites reside (Zhang et al., 2021; Reid et al., 2020; Hartmann et al., 2021; Becker et al., 2023). To analyse the impact of the LEL on the ion binding, we used a KCC2_2-4-2_ chimera, in which the LEL is from mouse KCC4 is exchanged with the LEL of rat KCC2b (Hartmann et al., 2010). We introduced six single mutations into the KCC2_2-4-2_ chimera, which differ in the ion coordination between KCC2 and KCC4 (Hartmann et al., 2021; Becker et al., 2023). These are ion binding residues that are not functionally relevant in *mm*KCC4 (I^132S^, P^429H^, and I^434E^), but in *rn*KCC2b (Hartmann et al., 2021; Becker et al., 2023) and ion binding residues, in which mutations lead to a greater reduction in transport activity in *rn*KCC2b than in *mm*KCC4 (T^432A^, G^134A^ and G^433A^; annotation according to *mm*KCC4 (Becker et al., 2023). Therefore, we substitute the following three K^+^ binding sites in the KCC2_2-4-2_ chimera, which results in: KCC2_2-4-2_^I111S^, KCC2_2-4-2_^P409H^ and KCC2_2-4-2_^T412A^ (annotation according to *rn*KCC2b). We also substitute Cl^−^ binding sites in the KCC2_2-4-2_ chimera, which results in: KCC2_2-4-2_^G113A^ (Cl_1_) and KCC2_2-4-2_^G413A^ and KCC2_2-4-2_^I414E^ (Cl_2_). All of the mutants showed transfection rates in HEK293 cells equal to that of KCC2_2-4-2_ (Figure 6B). The K^+^ binding site mutant KCC2_2-4-2_^I111S^ (248% ± 44%, p = 0.0002) enhanced activity, whereas the mutant KCC2_2-4-2_^T412A^ (60% ± 17%, p = 1.38 × 10^−6^) resulted in diminished activity compared to KCC2_2-4-2_ (100% ± 10%) (Figure 6A; Table 4). KCC2_2-4-2_^P409H^ (33% ± 12%, p = 0.0086) resulted in abolished transport activity compared to mock transfected cells (25% ± 9%). The Cl_1_ site mutant KCC2_2-4-2_^G113A^ (34% ± 17%, p = 2.79 × 10^−14^) and the Cl_2_ site mutant KCC2_2-4-2_^I414E^ (40% ± 10%, p = 6.05 × 10^−14^) resulted in diminished transport activity compared to KCC2_2-4-2_ (100% ± 10%), whereas the Cl_2_ site mutant KCC2_2-4-2_^G413A^ (26% ± 8%, p = 0.46) resulted in abolished transport activities compared to mock transfected cells (25% ± 9%) (Figure 6A; Table 4).

**TABLE 4:** Transport activity of chosen binding site mutants in KCC2_2-4-2_ chimera.

Construct	Mean ± SD	Significance in comparison to KCC2b_2-4-2_	Significance in comparison to mock
mock	25% ± 9%	***	-
KCC2b_2-4-2_	100% ± 10%	-	***
I111S	248% ± 44%	***	***
P409H	33% ± 12%	***	n.s
T412A	60% ± 17%	***	***
G113A	34% ± 7%	***	***
G413A	26% ± 8%	***	n.s.
I414E	40% ± 10%	***	***

p ≥ 0.01: n.s., p < 0.05: **, p < 0.001: ***.

To analyse whether the ion coordination site substitutions affect the transport activity differently in the KCC2_2-4-2_ chimera compared to *rn*KCC2b^WT^ (Becker et al., 2023), the mutants were measured simultaneously in both backgrounds (Supplementary Figures 1, 2; Supplementary Tables 3). All of the mutants showed transfection rates in HEK293 cells equal to the background construct (Supplementary Figures 1B, 2B). For comparison the KCC2_2-4-2_ constructs have been normalized according to *rn*KCC2b^WT^ *,* since *rn*KCC2^WT^ (100% ± 7%) and KCC2_2-4-2_ (102% ± 22%, p = 0.55) have similar transport activities (Supplementary Figures 1A, 2A; Supplementary Table 3). Next, wee compared the transport activities of the mutants present in the *rn*KCC2b and KCC2_2-4-2_ chimeric backgrounds. The transport activities of the mutants P^409H^, T^412A^, G^113A^, G^413A^, and I^414E^ in the *rn*KCC2b^WT^ compared to the same mutants in the KCC2_2-4-2_ chimeric background revealed no significant differences. In brief, the transport activity of P^409H^ in *rn*KCC2b (43% ± 9%) and KCC2_2-4-2_ (33% ± 11%, p = 0.018), of T^412A^ in *rn*KCC2b (59% ± 5%) and KCC2_2-4-2_ (64% ± 8%, p = 0.017), of G^113A^ in *rn*KCC2b (43% ± 11%) and KCC2_2-4-2_ (33% ± 5%, p = 0.016), of G^413A^ in *rn*KCC2b (27% ± 6%) and KCC2_2-4-2_ (26% ± 8%, p = 0.9), and of I^414E^ in *rn*KCC2b (37% ± 9%) and KCC2_2-4-2_ (37% ± 10%, p = 0.95) are similar (Supplementary Figures 1A, 2A; Supplementary Table 3).

However, a significant difference between the I^111S^ mutant in the *rn*KCC2b background (70% ± 9%) compared to the chimeric background (271% ± 57%, p = 0.0002) is present (Supplementary Figure 1A; Supplementary Table 3). Thus, the different relevance of the potassium binding sites P^409^ and T^412^ and the chloride binding sites in Cl_1_ G^113^ and Cl_2_ G^413^ and I^414^ cannot be attributed to the different structures of the LEL. However, the different structure of the LEL does have an influence on potassium coordination in I^111^.

## Discussion

Recently published CryoEM structures revealed that the ion binding sites in the CCCs are highly conserved (Chi et al., 2021; Chew et al., 2021; Zhang et al., 2021; Reid et al., 2020; Xie et al., 2020; Chew et al., 2019; Liu et al., 2019). KCCs have one potassium coordination site and two chloride coordination sites, although KCCs transport both ions in a 1:1 stoichiometry (Zhang et al., 2021; Reid et al., 2020; Liu et al., 2019). Mutation of residues coordinating potassium in KCC1 (tyrosine in TM3), KCC2 (asparagine and isoleucine in TM1, tyrosine in TM3, and proline and threonine in TM6), KCC3 (tyrosine in TM3 and threonine in TM6), KCC4 (asparagine in TM1 and tyrosine in TM3), and NKCC1 (tyrosine in TM3 and proline and threonine in TM6) abolished or diminished transporter activity (Zhang et al., 2021; Reid et al., 2020; Chew et al., 2019; Liu et al., 2019; Hartmann et al., 2021). Mutation of residues that coordinate chloride in Cl_1_ (in KCC2: glycine, valine, and isoleucine in TM3) and chloride in Cl_2_ (in KCC2: glycine, valine, methionine in TM6 and tyrosine in TM10; in KCC1, KCC3, KCC4, NKCC1, and NCC: tyrosine in TM10) also lead to reduced or abolished transport activities (Zhang et al., 2021; Reid et al., 2020; Chew et al., 2019; Liu et al., 2019; Neumann et al., 2021; Becker et al., 2023; Nan et al., 2022). These results indicated that in KCC2 all ion coordination sites are essential and that both chloride binding sites are also functionally relevant (Hartmann et al., 2021; Becker et al., 2023).

**FIGURE 7:**
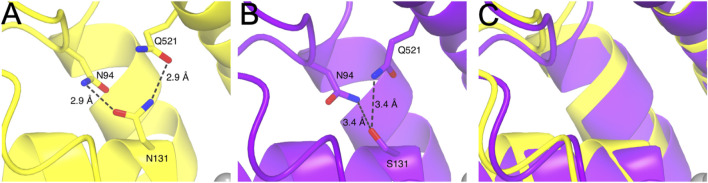
Structural depiction of *mm*KCC4^N131^, *mm*KCC4^N131S^ and superposition of experimental (PDB deposition: 7D99) and predicted AlphaFold3 model around the K^+^ binding site. **(A)** Structural representation of *mm*KCC4^N131^ shown in yellow. Intermolecular interactions of the side chain of N^131^ to N^94^ and Q^521^ are drawn as dashed lines with distances given in Ångström (Å). **(B)** Structural consequences for the *mm*KCC4^N131S^ variant obtained from AlphaFold3 prediction model. Dashed lines indicate atomic distances in Å. **(C)** Superposition of PDB entry 7D99 and predicted AlphaFold3 model for *mm*KCC4 ^N131S^ around the K^+^ binding site showing the structural similarity.

Our comprehensive analyses of the functional relevance of the ion binding sites in *mm*KCC4 have surprisingly revealed that not all highly conserved ion coordination sites are functionally relevant. Three (N^131^, Y^216^, and T^432^) out of five residues are necessary for K^+^ coordination in *mm*KCC4. A structural inspection demonstrates that N^131^ makes two hydrogen bonds via its side chain. The side chain’s amine group of N^131^ bridges to the side chain hydroxy group Q^521^, while the side chain hydroxy group of N^131^ forms a hydrogen bond to the amine group of the side chain from N^95^. Mutation of N^131^ to a serine in *mm*KCC4^N131S^ means that the amine-hydroxy hydrogen bond with Q^521^ is no longer possible. Therefore, it is possible that the backbone of *mm*KCC4 moves away from the K^+^ coordination centre due to the absence of these interactions, which results in a weaker coordination of the K^+^ ion and thus, a reduced transport activity is observed. To analyse this, we used AlphaFold3 to predict the structural consequences of *mm*KCC4^N131S^, however a backbone shift was not verifiable by the prediction (Figure 7). The residue Y^216^ coordinates the K^+^ ion via its side chain hydroxyl group. Evidently, replacing this amino acid, as observed in *mm*KCC4^Y216F^, destabilizes the coordination of the K^+^ ion and therefore, a significantly reduced transport activity is detectable. I^132^ and P^429^ coordinate K^+^ via their backbone oxygen. Thus, a substitution present in *mm*KCC4^I132S^ and *mm*KCC4^P429F^ will not lead to significant changes in transport rate. Structurally, T^432^ coordinates its ion via backbone interaction and side chain interaction with both oxygens. For *mm*KCC4^T432A^ the backbone interaction is still possible, however, the missing hydroxy group in the side chain of this mutation could reduce the binding strength of this coordination site.

All three residues in Cl_1_ (G^134^, V^135^, and I^136^) are functionally relevant for *mm*KCC4 and these residues coordinate the Cl^−^ ion with oxygen backbone interactions. Furthermore, three (G^433^, M^435^, and Y^589^) out of four residues in Cl_2_ are functionally important for chloride coordination. On a structural level, *mm*KCC4^G433A^ does not display any additional interactions, apart from the possibility for coordination of the Cl^−^ ion by its backbone oxygen. By the available structural data, the functional consequences of this mutation cannot be explained. Therefore, we used AlphaFold3 to determine the structural consequences of the mutant *mm*KCC4^G433A^. Unfortunately, further insights were not revealed and the binding of all three ions in the predicted model are at the same location as observed for PDB deposition 7D99. *mm*KCC4^M435Q^ coordinates the chloride anion via the oxygen of the backbone, nevertheless, the side chain of M^435^ shows an S-aromatic interaction with the residue F^205^. The sulphur atom of M^435^ is around 5 Å far away from the centre of the ring of F^205^ and due to the absence of this interaction in *mm*KCC4^M435Q^, this could result in a higher degree of flexibility. Therefore, a backbone shift could occur which weakens the interaction of this amino acid with the anion in Cl_2_. The mutant *mm*KCC4^Y589F^ cannot coordinate Cl^−^ anion anymore, because the hydroxy group of the side chain ring is absent in this mutation, which results in the significantly reduced transport rate. *mm*KCC4^I434E^ appears to have no significant changes in the transport rate as the coordination of the Cl^−^ ion can still occur through the backbone’s oxygen. In summary, the potassium and the two chloride binding sites in *mm*KCC4 are functionally relevant. However, fewer amino acid sites are required for the coordination of the ions than for *rn*KCC2b. This indicates that the coordination of the ion binding sites in *rn*KCC2b is much more stringent than in *mm*KCC4. This different stringency of ion coordination may be an explanation for the different ion affinities among CCCs.

What might be the reasons for the different relevance of the ion coordination sites between KCC2 and KCC4? Previous studies have shown that the extracellular loops have an indirect effect on the coordination of ion binding sites (Hartmann et al., 2021; Becker et al., 2023; Hartmann et al., 2010). Artificial elongation of the second extracellular loop (EL2) by a 37-aa-long HA tag resulted in a shift of the potassium coordination site in KCC2 (Hartmann et al., 2021; Becker et al., 2023), which is probably due to the EL2 moving into the interaction range of the LEL (Hartmann et al., 2021). Since the LEL connects TM5 and TM6, subtle conformational changes in the LEL have a direct effect on the conformation of TM6 (Reid et al., 2020). TM6, in turn, harbors potassium (proline and threonine) and chloride coordination sites in Cl_2_ (Figures 1, 2) (glycine, isoleucine, and methionine) (Hartmann et al., 2021; Becker et al., 2023), that might be then affected.

As the LEL of the KCCs is organised differently and the structural requirements have different effects on the function of the transporters (Reid et al., 2020; Xie et al., 2020; Hartmann et al., 2010; Chi et al., 2020), we have investigated here to what extent an exchange of the LEL might influences ion coordination. Therefore, we used the KCC2_2-4-2_ chimera, in which the LEL of *rn*KCC2b is exchanged with the one of *mm*KCC4. We then substituted ion coordination sites, that differ in the relevance of ion coordination sites between *rn*KCC2b and *mm*KCC4. These ion coordination sites are located in TM1 (K^+^ site: I^111^ and Cl_1_ site: G^113^) and TM6 (K^+^ sites: P^409^ and T^412^; Cl_2_ sites: G^413^ and I^414^). Substitution of residues located in TM6 within the chimera lead to the same decrease in transport activity as shown in *rn*KCC2b. Thus, the different structure of the LEL does not lead to such strong conformational changes within the TM6, so that the coordination of the ions is affected. The situation is different with K^+^ coordination site I^111^. Substitution of I^111^ in KCC2_2-4-2_ chimera lead to an enhanced transport activity, which is not consistent with the results in either *rn*KCC2b (decrease in activity (Hartmann et al., 2021) or *mm*KCC4 (no effect). I^111^ is located in TM1 (Zhang et al., 2021), that is not directly linked with the LEL. On a 3-dimensional level, the TM1 is coupled with TM6 through the coordination of the potassium ion (Figure 2) (Zhang et al., 2021; Hartmann et al., 2021; Becker et al., 2023). Therefore, conformational changes induced by the LEL would not primarily manifest themselves in changes in potassium coordination of TM6, but only through shifts in the coupling of potassium coordination sites in TM1. Thus, changes within the LEL can have an influence on ion coordination sites. However, the different relevance of the ion coordination sites between *rn*KCC2 and *mm*KCC4 cannot be attributed solely to the different structured LEL; other structural elements must also be involved.

These other structural elements could be surrounding TMs. Isenring and Forbush were able to show that additional amino acid sites in TM2, TM4, and TM7 are also responsible for the different ion affinities between human and shark NKCC1 (Delpire and Guo, 2020; Payne et al., 1995; Isenring et al., 1998c; Isenring and Forbush, 1997; Isenring and Forbush, 2001; Isenring et al., 1998b; Isenring et al., 1998a). Experiments with chimeras suggest that this is based on sequence differences in TMs, that are not directly involved in ion coordination, but may have a conformational effect (Delpire and Guo, 2020; Isenring et al., 1998c; Isenring and Forbush, 1997; Isenring and Forbush, 2001; Isenring et al., 1998b; Isenring et al., 1998a; Payne, 2012). In brief, evolutionary variable residues in TM2 were shown to influence Na^+^ and K^+^ kinetics, in TM4 K^+^ and Cl^−^ kinetics, and TM7 Na^+^, K^+^ and Cl^−^ kinetics (Delpire and Guo, 2020; Payne et al., 1995; Isenring et al., 1998c; Isenring and Forbush, 1997; Isenring and Forbush, 2001; Isenring et al., 1998b; Isenring et al., 1998a). There are also numerous different residues in surrounding TMs among KCCs, which may also have an additional effect on ion coordination (Figure 1). Future experiments are needed to demonstrate to what extent residues in the surrounding TMs also have an effect on ion coordination.

In summary, our experiments revealed that not all ion coordination sites detected by CryoEM are relevant for ion coordination in *mm*KCC4. Thus, there is a certain flexibility in the coordination of the ions, which might be decisive for the different ion affinities among CCCs. The structural reason for the different relevance of the ion coordination sites is partly due to the different organisation of the LEL, but other structural elements might also play a role here.

## Data Availability

The datasets presented in this study can be found in online repositories. The names of the repository/repositories and accession number(s) can be found in the article/Supplementary Material.
