# Supplementary figures and images for: Highly conserved ion binding sites are not all functionally relevant in mouse KCC4
Source: Front Mol Biosci. 2025 Mar 31;12:1556250. doi: 10.3389/fmolb.2025.1556250 (PMC11994965; doi:10.3389/fmolb.2025.1556250)

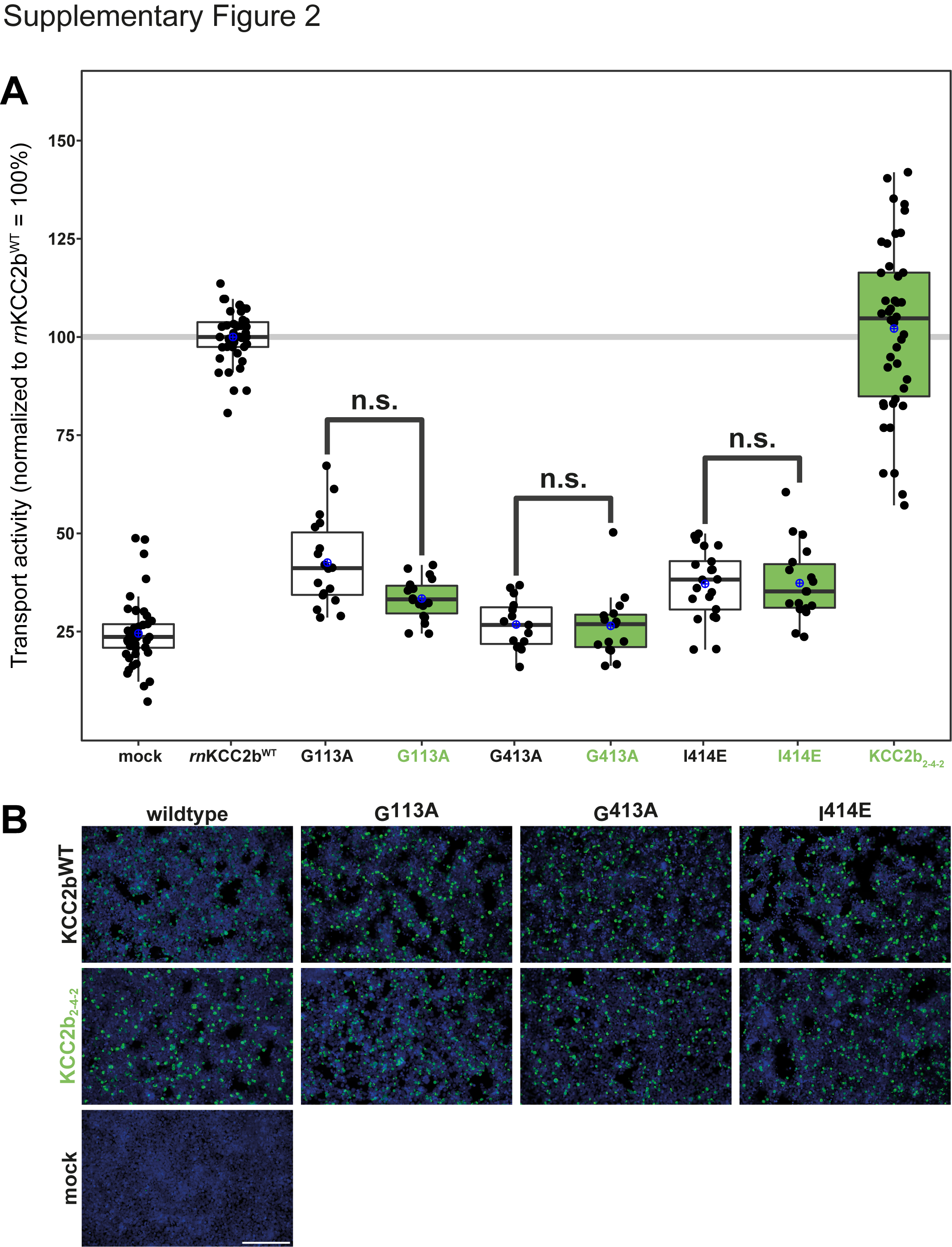

Supplement: Supplementary file 1 [file Image2.tif]

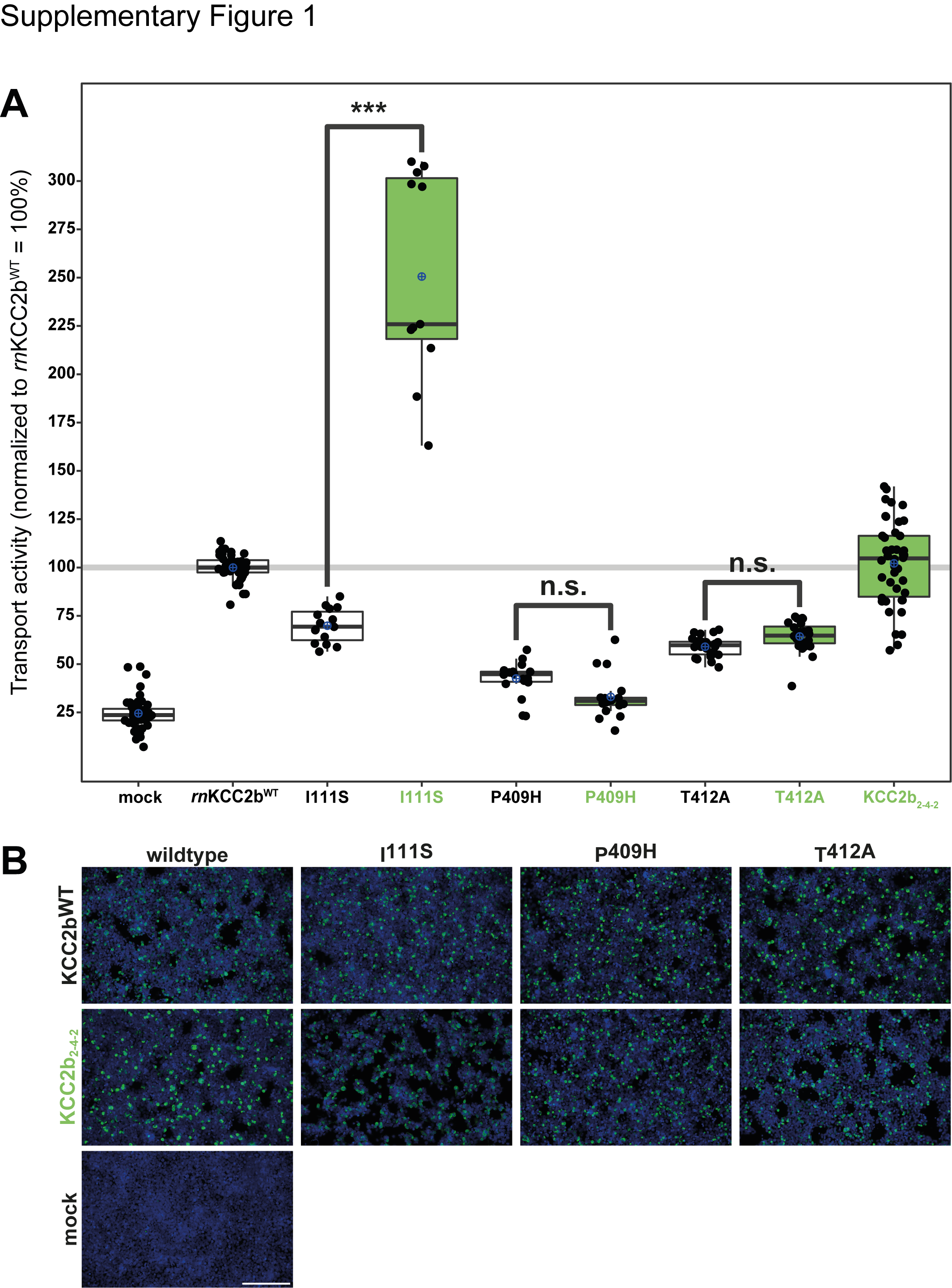

Supplement: Supplementary file 2 [file Image1.tif]
