# Supplementary material for: Highly conserved ion binding sites are not all functionally relevant in mouse KCC4
Source: Front Mol Biosci. 2025 Mar 31;12:1556250. doi: 10.3389/fmolb.2025.1556250 (PMC11994965; doi:10.3389/fmolb.2025.1556250)
Supplement: Supplementary file 3 [file DataSheet1.pdf]

Supplementary information

Supplementary Figure 1: Comparison of selected potassium binding site substitutions in *rn*KCC2b and KCC2$_{2\text{-}4\text{-}2}$ chimera

HEK293 cells were transiently transfected with *rn*KCC2b^{WT}, KCC2$_{2\text{-}4\text{-}2}$ chimera and *rn*KCC2b or KCC2$_{2\text{-}4\text{-}2}$ variants with mutations in the chosen potassium binding site residues. Cells were then seeded in parallel for Tl^+ flux measurements and immunocytochemistry. A) Tl^+ flux measurements were performed to determine the transport activities. *rn*KCC2b^{WT} (100% ± 7%) and KCC2$_{2\text{-}4\text{-}2}$ (102% ± 22%, p=0.55) do not differ in their activities. The constructs originating from *rn*KCC2b are labeled in black and from KCC2$_{2\text{-}4\text{-}2}$in green. The transport activities of P^{409H} in *rn*KCC2b (43% ± 9%) and KCC2$_{2\text{-}4\text{-}2}$ (33% ± 11%, p= 0.018), of T^{412A} in *rn*KCC2b (59% ± 5%) and KCC2$_{2\text{-}4\text{-}2}$ (64% ± 8%, p= 0.017) are similar. Substitution of I^{111} to serine in *rn*KCC2b (70% ± 9%) significantly differs from substitution of the same mutation in KCC2$_{2\text{-}4\text{-}2}$ (271% ± 57%, p= 0.0002). The graph represents the data of at least five independent measurements, including three technical replicates, normalized to *rn*KCC2b^{WT}. Statistical analysis can be seen in Supplementary Table 3. B) Immunocytochemistry was used to monitor the transfection rate of the KCC2 variants (green) and cell staining by DAPI (blue). Representative immunocytochemical images were used for the biological replicates. The scale bar represents 200 µm.

Supplementary Figure 2: Comparison of selected chloride binding site substitutions in *rn*KCC2b and KCC2$_{2\text{-}4\text{-}2}$ chimera

HEK293 cells were transiently transfected with *rn*KCC2b^{WT}, KCC2$_{2\text{-}4\text{-}2}$ chimera, *rn*KCC2b and KCC2$_{2\text{-}4\text{-}2}$ variants with mutations in the chosen chloride binding site residues. Cells were then seeded in parallel for Tl^+ flux measurements and immunocytochemistry. A) Tl^+ flux measurements were performed to determine the transport activity. *rn*KCC2b (100% ± 7%) and KCC2$_{2\text{-}4\text{-}2}$ (102% ± 22%, p=0.55) do not differ in their activities. The constructs originating from *rn*KCC2b are labeled in black and from KCC2$_{2\text{-}4\text{-}2}$ in green. The transport activities of G^{113A} in *rn*KCC2b (43% ± 11%) and KCC2$_{2\text{-}4\text{-}2}$ (33% ± 5%, p= 0.016), of G^{413A} in *rn*KCC2b (27% ± 6%) and KCC2$_{2\text{-}4\text{-}2}$ (26% ± 8%, p= 0.9), and of I^{414E} in *rn*KCC2b (37% ± 9%) and KCC2$_{2\text{-}4\text{-}2}$ (37% ± 10%, p= 0.95) are similar. The graph represents the data of at least five independent measurements, including three technical replicates, normalized to *rn*KCC2b^{WT}. Statistical analysis can be seen in Supplementary Table 3. B) Immunocytochemistry was used to monitor the transfection rate of the KCC2 variants (green) and cell staining by DAPI (blue). Representative immunocytochemical images were used for the biological replicates. The scale bar represents 200 µm.

Supplementary Table 1: Forward primers used for site-directed mutagenesis in *mm*KCC4

Constructs	Sequence 5' to 3'
*mm*KCC4 N131S	CCGTGCCTGCAGTCC
*mm*KCC4 I132S	TGCCTGCAGAACTCCTTGGGTGTTATC
*mm*KCC4 Y216F	TTTGCAGGCGCCATGTTC
*mm*KCC4 P429H	GGCATCTACTTCCACTCTGTAACTGGG
*mm*KCC4 T432A	TACTTCCCCGTCTGTAGCT
*mm*KCC4 G134A	CAGAACATCTTGGCAGTTATCCTTTTC
*mm*KCC4 V135T	AACATCTTGGGTACTATCCTTTTCCTG
*mm*KCC4 I136E	ATCTTGGGTGTTGAACTTTTCCTGCGT
*mm*KCC4 G433A	CCGTCTGTAACTGCCATCATGGCAGGA
*mm*KCC4 I434E	TCTGTAACTGGGGAAATGGCAGGATCC
*mm*KCC4 M415Q	TAACTGGGATCCAGGCAGGATCCAA
*mm*KCC4 Y589F	TTCCTCATGTGCTTTATGTTCGTCAAT

Abbreviations used are as follows: *mm, mus musculus*

Supplementary Table 2: Forward primers used for site-directed mutagenesis in KCC2$_{2\text{-}4\text{-}2}$

Constructs	Sequence 5' to 3'
KCC2$_{2\text{-}4\text{-}2}$ I111S	CCGTGCCTGCAGAACAGCTTTGGTGTTATC
KCC2$_{2\text{-}4\text{-}2}$ P409H	GCATCTATTTCCACTCAGTCACAG
KCC2$_{2\text{-}4\text{-}2}$ T412A	CTATTTCCCCTCAGTCGCAGGGATCATGGCTG
KCC2$_{2\text{-}4\text{-}2}$ G113A	CAGAACATCTTTGCTGTTATCCTCTTT
KCC2$_{2\text{-}4\text{-}2}$ G413A	TCAGTCACAGCGATCATGGCT
KCC2$_{2\text{-}4\text{-}2}$ I414E	AGTCACAGGGGAAATGGCTGG

Supplementary Table 3: Transport activity of chosen binding site mutants in *rn*KCC2b^{WT} and KCC2$_{2\text{-}4\text{-}2}$ chimera

Constructs based on *rn*KCC2	Mean ± SD *rn*KCC2 constructs	Significance in comparison to *rn*KCC2b *mutant*	Mean ± SD KCC2$_{2\text{-}4\text{-}2}$ constructs	Constructs based on KCC2$_{2\text{-}4\text{-}2}$
mock	25% ± 9%	-		-
*rn*KCC2b^{WT}	100% ± 7%	n.s.	102% ± 22%	KCC2b$_{2\text{-}4\text{-}2}$
I111S	70% ± 9%	***	271% ± 57%	I111S
P409H	43% ± 9%	n.s.	33% ± 11%	P409H
T412A	59% ± 5%	n.s.	64% ± 8%	T412A
G113A	43% ± 11%	n.s.	33% ± 5%	G113A
G413A	27% ± 6%	n.s.	26% ± 8%	G413A
I414E	37% ± 9%	n.s.	37% ± 10%	I414E

Abbreviations used are as follows: *rn, rattus norvegicus,* $p \geq 0.01$: n.s., $p < 0.001$: ***
